# The Molecular Effect of Diagnostic Absorbed Doses from ^131^I on Papillary Thyroid Cancer Cells In Vitro

**DOI:** 10.3390/molecules22060993

**Published:** 2017-06-15

**Authors:** Mariusz Stasiołek, Zbigniew Adamczewski, Przemysław W. Śliwka, Bartosz Puła, Bolesław Karwowski, Anna Merecz-Sadowska, Marek Dedecjus, Andrzej Lewiński

**Affiliations:** 1 Department of Neurology, Polish Mother’s Memorial Hospital—Research Institute, 93-338 Lodz, Poland; mstasiolek@yahoo.de; 2 Department of Endocrinology and Metabolic Diseases, Medical University of Lodz, 93-338 Lodz; Poland; zbadam@o2.pl; 3 Department of Endocrinology and Metabolic Diseases, Polish Mother’s Memorial Hospital—Research Institute, 93-338 Lodz, Poland; p.sliwka87@gmail.com; 4 Department of Hematology, Institute of Hematology and Transfusion Medicine, 02-776 Warsaw, Poland; bartosz.pula@gmail.com; 5 Food Science Department, Medical University of Lodz, 90-151 Lodz, Poland; boleslaw.karwowski@umed.lodz.pl (B.K.); anna.merecz@umed.lodz.pl (A.M.-S.); 6 Department of Oncological Endocrinology and Nuclear Medicine, Maria Skłodowska-Curie Memorial Cancer Center and Institute of Oncology, 02-781 Warsaw, Poland; marek.dedecjus@gmail.com

**Keywords:** ^131^I, genetic material stability, DNA damage, thyroid stunning, papillary thyroid carcinoma

## Abstract

Diagnostic whole-body scan is a standard procedure in patients with thyroid cancer prior to the application of a therapeutic dose of ^131^I. Unfortunately, administration of the radioisotope in a diagnostic dose may decrease further radioiodine uptake—the phenomenon called “thyroid stunning”. We estimated radiation absorbed dose-dependent changes in genetic material, in particular in the sodium iodide symporter (NIS) gene promoter, and the NIS protein level in a K1 cell line derived from the metastasis of a human papillary thyroid carcinoma exposed to ^131^I in culture. The different activities applied were calculated to result in absorbed doses of 5, 10 and 20 Gy. Radioiodine did not affect the expression of the *NIS* gene at the mRNA level, however, we observed significant changes in the NIS protein level in K1 cells. The decrease of the NIS protein level observed in the cells subjected to the lowest absorbed dose was paralleled by a significant increase in 8-oxo-dG concentrations (*p* < 0.01) and followed by late activation of the DNA repair pathways. Our findings suggest that the impact of ^131^I radiation on thyroid cells, in the range compared to doses absorbed during diagnostic procedures, is not linear and depends on various factors including the cellular components of thyroid pathology.

## 1. Introduction

Thyroid cancer represents the most frequent endocrine malignancy, with differentiated papillary thyroid carcinomas (PTC) being the most common variant, occurring in about 80–90% of all cases.

The unique features of differentiated thyroid cancer (DTC) cells are: ability to take up iodine, presence of the thyroid-stimulating hormone (TSH) receptor, synthesis of thyroglobulin (Tg) and, in some cases, synthesis of thyroid hormones. These characteristics of DTC enable the use of radioactive iodine, especially ^131^I, in diagnostic and therapeutic procedures in this particular disease. Physical characteristics of this isotope, particularly the emission of gamma and beta radiation, allow for the use of ^131^I for diagnosis as well as therapy.

Diagnostic whole-body scan (DxWBS) is a standard procedure in patients with thyroid cancer prior to the application of a therapeutic dose of ^131^I [1]. Unfortunately, literature data indicate that administration of a radioisotope in a diagnostic dose may decrease further the radioiodine uptake—the phenomenon called “thyroid stunning”. It was first described in 1951, and in the next years provoked numerous attempts to confirm its existence and/or clarify the underlying molecular mechanisms [2,3,4,5,6,7,8,9,10,11,12,13,14]. The potential occurrence of “thyroid stunning” is especially undesirable in the case of thyroid tumor metastasis, where an insufficient radioiodine therapeutic effect may result in regrowth and further spread of cancer cells. Park et al. [2] reported that the stunning effect was not observed in patients when diagnostic dose was reduced to 37–74 MBq. Avram et al. had similar observations while conducting DxWBS 24 h after administration of 37 MBq of ^131^I, where he proved diagnostic effectiveness of this procedure, demonstrating 92% concordance of DxWBS with the post-therapy whole body scan (RxWBS) in 303 patients [3]. Their results did not confirm previous observations of Verburg et al. [4] who indicated that ablation therapy was less effective if 40 MBq ^131^l was to be used during diagnostic procedures. Difficulties in evaluating the stunning phenomenon occur in accordance with clinical observations indicating a possible existence of an early effect of therapeutic activities on RxWBS imaging. The decrease of radioiodine concentration in RxWBS is presumed to be primarily the consequence of destructive effects of the therapeutic dose of ^131^I [5]. However, observations from radioiodine treatment of hyperthyroidism cases indicate that the radiosensitivity of thyrocytes depended strongly on the nature of the underlying thyroid disease [15]. Moreover, ^131^I uptake in malignant thyroid tissue is almost always much lower than that in normal thyroid tissue [16]. In light of above-mentioned observations, it has to be considered that, due to the frequently coexisting pathologies, other than the tumor pathology of the thyroid, the assessment of radiosensitivity of target tissue is always to some extent imprecise, and can be calculated with approximation only [17].

Although several attempts have been made to explain the molecular mechanism of the stunning phenomenon, consistent and reproducible experimental data are lacking and there is still not enough information for creating the guidelines for diagnostic and therapeutic standards of radioiodine application in patients with DTC. The studies published so far have focused on characterization of the impact of ^131^I on the thyroid tissue remnants—unaffected normal follicular thyroid cells. This also applies to the in vitro studies mostly based on an assessment of the impact of ^131^I on porcine thyrocytes [18,19] and, in the case of our earlier study, on the freshly isolated human thyrocytes. To our knowledge, the influence of diagnostic doses of ^131^I on DTC cells have not been investigated on a molecular level, to date.

In the current study, we used the K1 cell line derived from the metastasis of human PTC [20] to characterize molecular events (in particular associated with NIS expression) occurring in response to different absorbed doses of radioiodine. Profiling assays screening through various PTC cell lines proved K1 cells, as well as BCPAP, KTC-1, and TPC-1 cell lines, to be viable models to study thyroid cancer [21]. Due to the very specific characteristics of our experiments, we chose the K1 cell line as the most universal model that has been commonly used in in vitro studies to evaluate the response of tumors to chemotherapy and radiotherapy. It is characterized by presence of the sodium-iodide symporter (NIS), thyroid peroxidase (TPO), and Tg, which are associated with the limited, but preserved, iodine uptake of these cells.

## 2. Results

In order to gain insight into possible cellular and molecular mechanisms underlying the stunning phenomenon, we performed a panel of experiments on cultured K1 cells exposed to various absorbed doses of ^131^I in vitro.

### 2.1. Apoptosis

**Figure 1:**
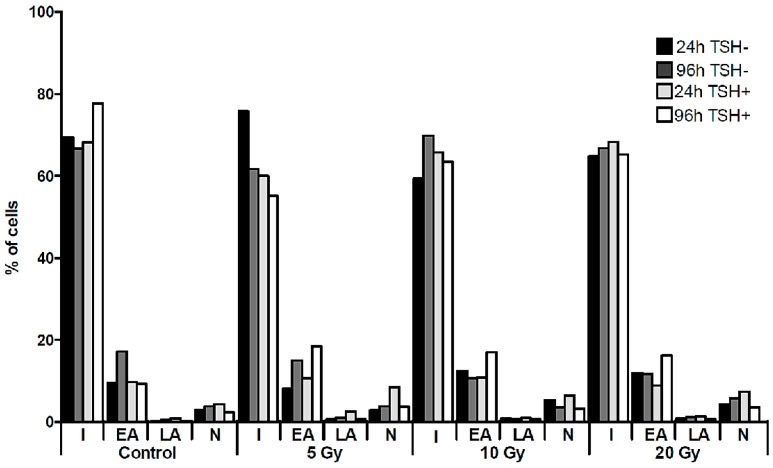
Cell death of K1 cells in culture with ^131^I. The graph presents the percentages of K1 cells undergoing apoptotic or necrotic death processes, as assessed by flow cytometry directly after 24 h of ^131^I exposure (5, 10, 20 Gy) or after additional 72 h of culture without ^131^I. The culture was performed parallel with or without TSH stimulation. I—intact cells, EA—early apoptosis, LA—late apoptosis, N—necrosis.

**Figure 2:**
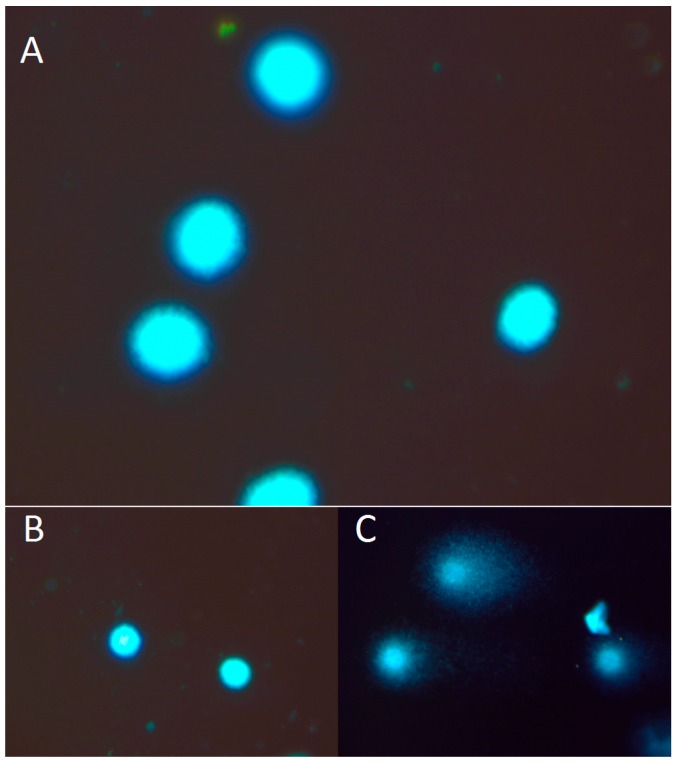
Representative images of DNA comets, obtained from K1 cells. (**A**) The picture shows intact DNA heads without the DNA tail. (**B**) Negative control. (**C**) Positive control (treated with 15 µM H_2_O_2_). The K1 cells were stained with 4′,6-diamidino-2-phenylindole (DAPI), observed in fluorescent microscopy at magnification 400×. DNA damage was calculated as the DNA tail area/whole DNA area (%) and the comet tail length (from the center of DNA head to the end of the DNA tail).

The intensity of the cell death processes of cultured cells was assessed directly after 24 and 96 h of culture with flow cytometry (FACS) (on the basis of Annexin V and Propidium Iodide staining) and by COMET assay. FACS analysis revealed that up to 80% of the cultured K1 cells remained intact without signs of apoptotic or necrotic death processes at every time point, regardless of the presence of TSH (Figure 1). Moreover, we did not observe any influence of the applied absorbed doses of ^131^I on the rate of apoptosis and necrosis of K1 cells in vitro. The confirmation of the FACS results was obtained in parallel experiments by COMET assay analysis. In all indicated time points and culture conditions, obtained images showed round, tight heads of DNA comets without signs of fragmentation, characteristic for apoptotic process (Figure 2). The low level of apoptotic and necrotic cell death confirmed that the applied experimental settings preserved viable cells for the molecular analyses in the study. In order to reproduce as close as possible the local microenvironment in vivo, all the further in vitro experiments were performed under TSH stimulation.

### 2.2. Expression of NIS Gene

**Figure 3:**
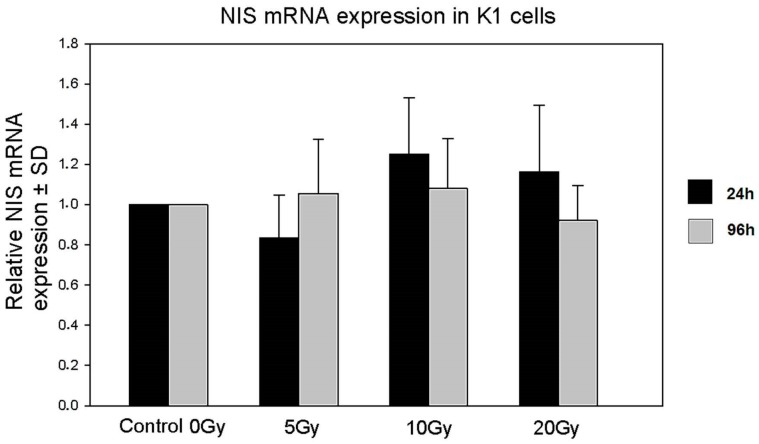
Expression of the *NIS* gene by K1 cells in culture with ^131^I. The graph shows relative NIS gene expression (±SD) in K1 cells directly after 24 h of ^131^I exposure (5, 10, 20 Gy) or after additional 72 h of culture without ^131^I.

We used the RT-qPCR technique to measure the influence of beta and gamma radiation emitted by ^131^I on *NIS* gene expression. Absorbed doses ranging from 5 to 20 Gy did not deregulate the expression of the *NIS* gene in cultured K1 cells (Figure 3).

### 2.3. Expression of the NIS Protein

**Figure 4:**
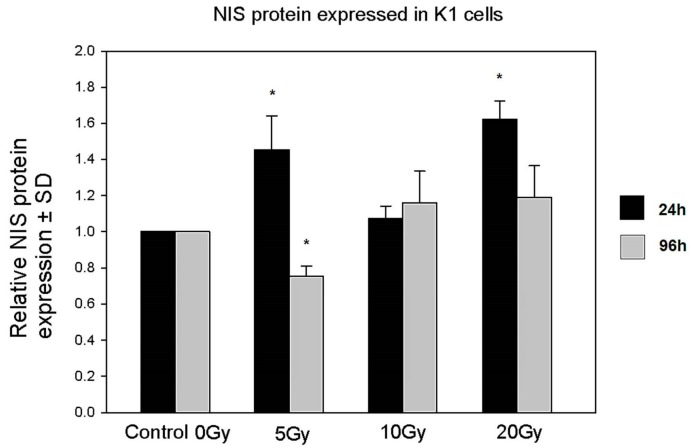
Expression of the NIS protein in K1 cells in culture. The graph shows relative NIS protein expression (±SD) obtained from cultured K1 cells directly after 24 h of ^131^I exposure (5, 10, 20 Gy) or after additional 72 h of culture without ^131^I (* *p* < 0.05).

Although the mRNA expression of the NIS protein remained unaffected by ^131^I, as presented above, we found a time- and absorbed ^131^I dose-dependent deregulation of NIS protein expression in K1 cells. Interestingly, cells analyzed after 24 h of incubation with ^131^I showed statistically significant up-regulation of NIS in the groups of the lowest (5 Gy) and highest (20 Gy) absorbed doses of radioiodine. This up-regulation was, however, reversed after 72 h of culture without ^131^I. The effect was most prominent in the 5 Gy group, where we observed significant down-regulation of NIS protein expression as compared to control untreated cells (Figure 4).

### 2.4. DNA Damage

#### 2.4.1. 8-Oxo-7,8-dihydro-2′deoxyguanosine (8-Oxo-dG)

**Figure 5:**
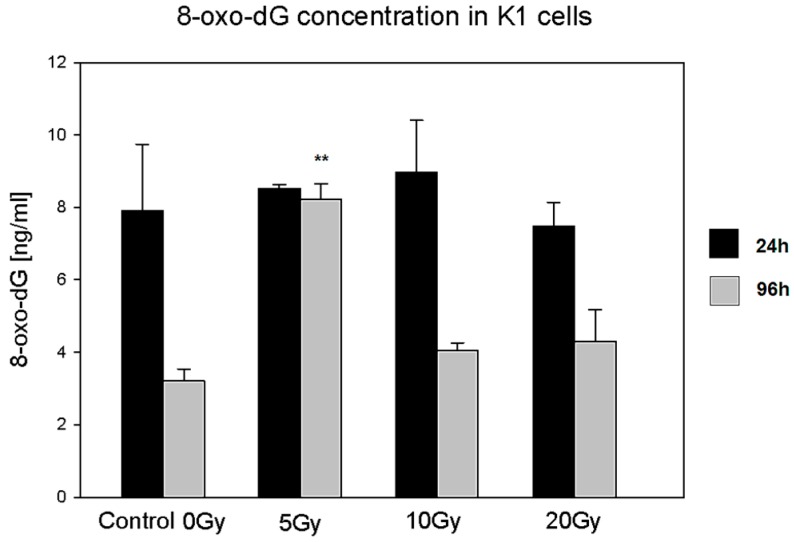
8-Oxo-dG concentration in K1 cells cultured with ^131^I. The graph presents 8-oxo-dG concentration (ng/mL ± SD) in cultured K1 cells directly after 24 h of ^131^I exposure (5, 10, 20 Gy) or after additional 72 h of culture without ^131^I (** *p* < 0.01).

8-oxo-dG is one of the most important DNA damage markers and can be considered as an indicator of cell condition in the case of radiation injury [22]. In our experiments, we observed statistically significant changes in the 8-oxo-dG level in the case of cells treated with the lowest dose of ^131^I (5 Gy) as compared to control untreated cells. The concentration of 8-oxo-dG was increased only at the 96 h time point indicating a prolonged accumulation of DNA damages even after the termination of ^131^I irradiation. (Figure 5).

#### 2.4.2. Apurinic/Apyrimidinic Sites (AP-Site)

**Figure 6:**
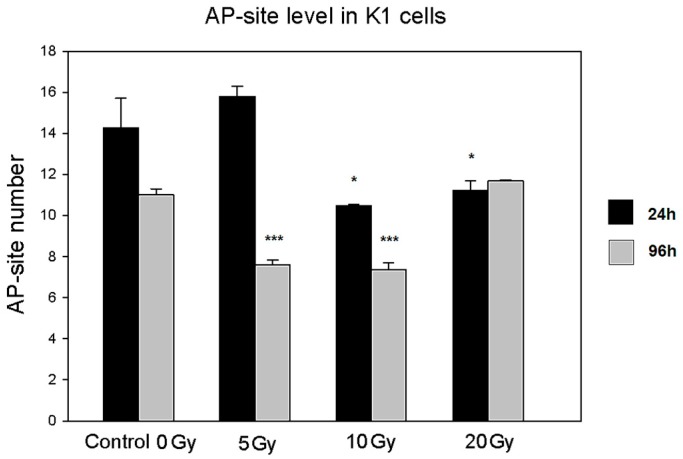
AP-site level in K1 cells cultured with ^131^I. The graph presents AP-site level (AP-sites/100,000 bp ± SD) in DNA measured in samples obtained from cultured K1 cells directly after 24 h of ^131^I exposure (5, 10, and 20 Gy) or after additional 72 h of culture without ^131^I (* *p* < 0.05; *** *p* < 0.001).

The number of AP-sites is considered to correlate with the intensity of the DNA repair process. In our experiments, we observed a time-dependent pattern of changes in AP-site level. At the early 24 h time point the level of AP-site decreased in cells exposed to middle and high absorbed doses of ^131^I, whereas at the late time point, a similar change was observed with low and middle doses of irradiation. While K1 cells are most likely characterized by a not fully functional DNA repair mechanism, these results may reflect the time- and irradiation dose-dependent activation of DNA repair pathways (Figure 6).

## 3. Discussion

The mechanisms underlying the phenomenon of so-called thyroid stunning, the reduction in iodine uptake after a diagnostic iodine scan, are still controversial.

In the current study, we used an experimental model to assess the effects of ^131^I on a K1 cell line derived from a metastasis of a well-differentiated PTC [20]. The applied experimental settings were analogous to that developed in our previous study [23] assessing the effects of the possible “stunning phenomenon” mechanisms in normal, non-cancerous cultured human thyrocytes under conditions close to those of the local thyroid microenvironment in vivo (e.g., TSH level, ^131^I absorbed doses). Moreover, in our experimental model, we took into consideration the fact that the inconsistency of clinical observations on thyroid stunning may come from evaluation of the influence of ^131^I diagnostic activity (range 37–370 MBq), rather than absorbed dose [2,3,4,5,6,7,8]. The biological effect of radioiodine after administration of standardized diagnostic activity of 111 MBq will vary depending on patients’ thyroid remnants and metastatic tissue. According to our observations, the patients who were qualified for radioiodine remnant ablation would absorb drastically different ^131^I doses ranging from 2.5 to 870 Gy after an administration of 111 MBq [24].

In order to adjust the conditions as much as possible to the clinical situation, doses of ^131^I applied in our study (ranging from 5 to 20 Gy) were similar to those absorbed in most cases during DxWBS by target cells and did not reach therapeutic doses. In accordance, cultured cells in our experiments retained high viability as no increase in apoptosis or necrosis was observed. Although the applied range of doses was proved in other studies to diminish iodine transport in experimental settings [11,19], in our experiments radioiodine did not affect the expression of the *NIS* gene at the mRNA level. However, we observed an increase in the NIS protein level in K1 cells after 24 h of incubation with ^131^I which reached statistical significance in the case of the lowest (5 Gy) and highest (20 Gy) absorbed doses, as compared to control untreated cells. A further 72 h of culture in medium devoid of ^131^I resulted in the significant down-regulation of the NIS protein in the K1 cells exposed previously to the lowest absorbed dose of ^131^I, whereas in the other experimental groups NIS protein expression returned to control values. It is known that post-translational modifications of proteins in cells irradiated with even small doses of ionizing radiation may be affected on the levels of phosphorylation, acetylation, and degradation [25]. Unfortunately, we were not able to extensively investigate the problem by further molecular analysis. The observed fluctuation of NIS protein levels without any changes in mRNA expression may be explained at least partially by the influence of irradiation on cap-dependent translation mechanisms resulting in the disturbances in protein synthesis (discussed in [25]).

Most interestingly, the decrease in the NIS protein level observed in the cells subjected to the lowest absorbed dose was paralleled by a significant increase in 8-oxo-dG concentration. This effect, specific for the lowest absorbed dose of ^131^I, may be a result of inefficiency in the cell defense and repair mechanisms, which could have been more effectively stimulated and compensated only in the K1 cells exposed to higher absorbed doses of radioiodine. Accordingly, it was demonstrated in various cancer cell lines, that DNA damage induced by low doses of irradiation did not activate the DNA damage sensor and failed to activate cell repair pathways [26]. We may assume that in our experimental settings, the basal activity of cell defense mechanisms was sufficient to maintain cell homeostasis during the first 24 h of culture. However, at further time points, the lack of activation of repair mechanisms in the lowest ^131^I dose group resulted in the accumulation of 8-oxo-dG. This assumption seems to be confirmed by the analysis of AP-site content in cultured cells. The number of AP-sites is considered to correlate with the intensity of the DNA repair process, based on removal of damaged or inappropriate bases by DNA *N*-glycosylases [27,28]. While ^131^I doses of 10 Gy and 20 Gy triggered protection mechanisms in K1 cells during initial 24 h of culture, the DNA repair pathways in the K1 cells exposed to the lowest ^131^I absorbed dose seemed to be activated later and detected first after further 72 h of culture—possibly in association with the observed accumulation of 8-oxo-dG. Whereas in the experiments with the highest ^131^I absorbed dose (20 Gy), the damage-repair process seemed to be already stabilized at the end of the culture.

Further studies of other factors that may affect iodine retention in PTC cells are required. It was shown that decreased expression of certain thyroid-specific transcription factors, mainly TTF-1 and Pax-8, led to down-regulation of proteins engaged in iodine metabolism. TTF-1 and Pax-8 bind to specific sites on the Tg, TPO, and NIS promoters [29]. Future analysis of these factors in the analogous model may enhance the collected information about possible mechanisms of stunning.

Our findings show that the impact of radiation on living cells, in the range analogous to doses absorbed during diagnostic procedures in patients, does not show the linear dose-effect. The results imply that the biological effect of ^131^I-associated radiation is an outcome of very complex, simultaneously ongoing processes of damage and repair in cancer cells. Additionally, our observations obtained in normal and malignant cells confirm that the effect of radiation may be dependent not only on the radiation-resistance of specific tissues, but also on coexisting thyroid pathology [23]. Despite all of the previously mentioned restrictions, scintigraphy using low ^131^I activity (e.g., 37 MBq) remains one of the best evaluation techniques for completion of post-operative staging of DTC. In conjunction with stimulated Tg levels, diagnostic ^131^I scans are used for identification of regional and distant metastatic disease and ^131^I therapy planning [30].

Therefore, the importance of understanding the molecular mechanisms of the thyroid stunning phenomenon is particularly important in light of the current design of therapy of chronic cancer patients with PTC metastasis or/and local recurrence, which consist of repeated whole body scans, followed by administration of therapeutic doses of radioiodine [31]. This is a prerequisite for searching for alternative individual diagnostic algorithms for qualification of DTC patients for ^131^I therapy that does not have a negative influence on the therapy’s outcome.

## 4. Materials and Methods

### 4.1. Cell Culture

The K1 PTC cell line was purchased from Sigma-Aldrich (Saint Louis, MO, USA). The cell line was maintained in culture medium containing RPMI 1640 with l-glutamine (Life Technologies, Carlsbad, CA, USA), enriched with 5% fetal calf serum (Sigma-Aldrich, Saint Louis, MO, USA), and supplemented with: Gibco^®^ Antibiotic-Antimycotic solution (contains 10,000 units/mL of penicillin, 10,000 µg/mL of streptomycin, and 25 µg/mL of Fungizone^®^ (Life Technologies)), kanamycin (100 µg/mL), transferrin (6 µg/mL), human recombinant insulin (10 µg/mL), somatostatin (10 ng/mL) and Gly His Lys (10 ng/mL) (all from Sigma-Aldrich, Saint Louis, MO, USA).

Cells from passages 2–5 were used in analysis. Cells were seeded in 6-well plates at a density of 1 × 10^6^ cell per well and were cultured in humidified atmosphere (5% CO_2_) at 37 °C in a Heraeus Type UT6 incubator (Kendro Laboratory Products, Hamburg, Germany). The cell culture was performed in the presence of ^131^I applied in various doses encompassing values expected in diagnostic applications (range of absorbed energy dose: 5–20 Gy). The influence of thyrotropin (thyroid-stimulating hormone, TSH) on analyzed parameters was evaluated in cultures supplemented with bovine pituitary TSH (Sigma-Aldrich, Saint Louis, MO, USA) (30 µU/mL). After 24 h, cells were harvested and divided for the direct analysis or further culture in fresh medium without ^131^I (cumulative culture time 96 h). In parallel control experiments, K1 cells underwent exactly the same culture procedures without ^131^I stimulation.

### 4.2. Fluorescence-Activated Cell Sorting (FACS) Analysis

Flow cytometry apoptosis analysis was performed with an Annexin V Apoptosis Detection Kit FITC Enzyme (eBioscience, San Diego, CA, USA) assay using Annexin V and Propidium iodide (PI) according to manufacturer’s protocol. 1 × 10^5^ cells in each sample were used for staining. Analysis was performed with FACSCanto II cytometer using FACSDiva software (version 6.1.2, BD Bioscience, Franklin Lakes, NJ, USA). At least 2 × 10^4^ cells were counted and the percentage of cells in the state of early apoptosis (EA), late apoptosis (LA), or necrosis (N) was measured.

### 4.3. RT-qPCR

Total RNA was extracted from cultured cells at the indicated time points with the QIAGEN RNeasy Mini Kit (Qiagen, Venlo, The Netherlands), according to the manufacturer’s protocol. UV spectrophotometry was used for quantification of isolated RNA. For each sample, cDNA was synthesized using random hexamers and a QuantiTect Reverse Transcription Kit (Qiagen). The relative quantitative PCR analysis was performed with a QuantiTect SYBR^®^ Green PCR Kit (Qiagen) and the Applied Biosystems^®^ 7500 Real-Time PCR System (Life Technologies). Oligonucleotide primers for the human NIS gene and for reference gene (GAPDH) were purchased from Metabion (Martinsried, Germany). Primer sequences were as follows: NIS 5′-TCTCTCAGTCAACGCCTCT-3′ (forward) and 5′-ATCCAGGATGGCCACTTCTT-3′ (reverse); GAPDH 5′-CACCTTCCCCATGGTGTCT-3′ (forward) and 5′-CCCCGGTTTCTATAAATTGAGC-3′ (reverse). All samples were done in triplets.

### 4.4. Comet Assay

The comet assay was carried out under alkaline conditions according to Singh et al. [32], with the following modifications. Cells were suspended in Low Melting Point (LMP) agarose (0.75% in PBS) and spread on microscope slides pre-coated with 0.5% normal agarose. Slides were then put in lysis solution (2.5 M NaCl, 0.1 M EDTA, 10 mM Tris and 1% Triton X-100, pH 10) for 1 h at 4 °C, and then incubated in an electrophoresis buffer (0.3 M NaOH and 1 mM EDTA, pH 13) for 20 min to allow unwinding of DNA. Electrophoresis was carried out for 20 min at 0.7 V/cm (30 mA). After electrophoresis, slides were washed in neutralization buffer (0.4 M Tris, pH 7.5), dried, stained with 2 µg/mL of a fluorescent stain—4′,6-diamidino-2-phenylindole (DAPI), and covered with a coverslip. Preparations were viewed and analyzed under 400× magnification. Images of comets for analysis were obtained using a JENOPTIK camera (Jenoptik, Jena, Germany), equipped with an UV filter block (excitation filter (359 nm) and barrier filter (461 nm)) connected to a fluorescent microscope (Delta Optical, Minsk Mazowiecki, Poland). Slides were scored using image analysis system, CaspLab v. 1.2.3β1 (University of Wroclaw, Institute of Theoretical Physics, Wroclaw, Poland) [33]. Measurements were made for 50 cells per analyzed slide after image archiving. The comet tail formation was analyzed as a quantitative measure of the DNA damage and the differences between cells were estimated on the basis of that parameter (percentages of damage in cell tail -control versus irradiated one). All the values in this study were expressed as mean ± standard error of mean (SEM). Differences between mean values were tested for using the One-Way ANOVA test. Appropriate negative and positive controls were made.

### 4.5. 8-Oxo-7,8-dihydro-2′deoxyguanosine (8-Oxo-dG) Measurement

Levels of 8-oxo-dG, a DNA damage biomarker, were determined in total DNA isolated from cultured K1 cells using OxiSelect™ Oxidative DNA Damage ELISA Kit (8-OHdG Quantitation) (Cell Biolabs, San Diego, CA, USA) according to the manufacturer’s protocol. Total DNA was isolated from cells with DNAzol (Life Technologies). UV spectrophotometry was used for quantification of isolated DNA. The extracted DNA was then denatured and cut with nuclease P1 (Sigma-Aldrich, Saint Louis, MO, USA), followed by incubation with alkaline phosphatase. The samples were then centrifuged (6000 rds/min, 5 min) and the supernatants were used for further analysis. Fifty microliters of sample or standards (0 to 20 ng/mL of 8-oxo-dG) were applied on 96-well plate, incubated 10 min at room temperature and then 50 µL of anti-8-oxo-dG antibody was added. After an enzymatic reaction, detection was performed in Multilabel Plate Reader Victor X (Perkin Elmer, Waltham, MA, USA). Optical density at 450 nm was measured and 8-oxo-dG concentration in each sample was calculated according to standards.

### 4.6. Apurinic/Apyrimidinic Sites (AP-Site) Determination

AP-sites were determined in total DNA isolated from cultured K1 cells using an OxiSelect™ Oxidative DNA Damage Quantitation Kit (Cell Biolabs) according to the manufacturer’s protocol. Total DNA was isolated as described previously. DNA samples were incubated with an aldehyde reactive probe (ARP) solution for 1 h at 37 °C. Next, 90 µL of TE buffer, 1 µL glycogen, 10 µL sodium acetate, and 300 µL of 98% ethanol were added, followed by 30 min incubation at −20 °C. After centrifugation, supernatants were removed and sediments were reconstituted in TE buffer. Fifty microliters of DNA solution in TE buffer (1 µg/mL) from each sample or standard DNA solution (0 to 40 AP-sites/100,000 base pairs) were applied on a 96-well plate, supplemented with 50 µL of binding buffer and incubated overnight at room temperature. After an enzymatic reaction, detection was performed in Multilabel Plate Reader Victor X (Perkin Elmer, Waltham, MA, USA). Optical density at 450 nm was measured and the number of AP-sites in each sample was calculated according to standards.

### 4.7. Enzyme-Linked Immunoabsorbent Assay (ELISA)

The NIS protein level was measured in lysates from cultured K1 cells using Enzyme-Linked Immunosorbent Assay Kit for Sodium-Iodide Symporter (USCN Life Science, Wuhan, China) according to manufacturer’s protocol. Harvested K1 cells were suspended in PBS at a density of 1.5 × 10^6^ cells/mL and frozen at −20 °C and thawed 3 times. Samples were then centrifuged at 1400 rds/min for 15 min. Supernatants were used in further analysis. One hundred microliters of each supernatant and standards (0 to 200 ng/mL) were placed on a 96-well plate and incubated for 2 h at 37 °C. Supernatants were next removed and 100 µL of reagent A for 1 h in 37 °C was applied. After the washing procedure, 100 µL of reagent B was applied for 30 min at 37 °C. After additional washing, 90 µL of substrate solution was added and samples were incubated 20 min at room temperature. Next, 50 µL of stopping solution was added and samples were analyzed in Multilabel Plate Reader Victor X (Perkin Elmer, Waltham, MA, USA). Optical density at 450 nm was measured and the NIS protein concentration in each sample was calculated according to standards.

### 4.8. Statistical Analysis

Statistical analysis was performed using Prism 5.0 (GraphPad, La Jolla, San Diego, CA, USA). In all the analyses, a two-tailed unpaired *t*-test was utilized and the results are presented as mean ± standard deviation (SD). Differences were considered significant for *p* < 0.05 in all the analyses.
